# Prospective Findings From the 1993 Pelotas (Brazil) Birth Cohort Study

**DOI:** 10.1016/j.jadohealth.2012.09.002

**Published:** 2012-12

**Authors:** Pedro C. Hallal, Charles E. Irwin

**Affiliations:** Postgraduate Program in Epidemiology, Federal University of Pelotas, Pelotas, Brazil; University of California, San Francisco, San Francisco, California

This supplement to the *Journal of Adolescent Health* is devoted to understanding the health and well-being of adolescents by taking the long view of this period of the life cycle. The articles herein analyze data collected from conception through age 15 from 4,500 individuals born in the city of Pelotas, Brazil, in 1993. The analyses provide us with not just a snapshot of adolescents' current health status, but rather an entire movie depicting the life-course trajectories and the emergence of health outcomes of this population during adolescence.

The first four articles of the supplement focus on early-life predictors of later health and behavior [1–4]. Wells et al show that infant weight and length gains were associated primarily with larger size in adolescence rather than increased adiposity, although later childhood gains in weight and height were predictors of fat mass [1]. In the second article, Martínez-Mesa et al report that maternal smoking during pregnancy negatively affects an offspring's height in adolescence [2]. The third and fourth articles report on the effect of socioeconomic change from birth to adolescence [3,4]. Hallal et al show that adolescents born to high-income families had half the odds of walking or cycling to school compared with those whose families became wealthy during adolescence, suggesting that the habit of commuting to school under one's own power was “built” early in life [3]. Anselmi et al examine the influence of poverty on mental health, showing that both persistent poverty and becoming poor over time were associated with a greater likelihood of developing conduct disorders in adolescence [4].

The next three articles focus on the health effects of behavioral changes during adolescence [5–7]. Menezes et al report a positive association between self-reported physical activity practice in adolescence and effort-dependent lung function, particularly among girls [5]. In contrast, Rombaldi et al show that too much time dedicated to physical activity (> 1,000 min/wk) may jeopardize school performance [6]. In addition, Noal et al report that the prevalence of wheezing at 15 years of age was 53% greater among obese versus nonobese adolescents [7].

The next three articles further explore environmental factors that influence obesity and perceptions of obesity from the perspective of parents and adolescents [8–10]. Assunção et al explore predictors of nutritional status change, finding that low-income girls were more likely than high-income girls to become obese from 11 to 15 years, and that high-income boys were more likely than low-income boys to cease being obese during adolescence [8]. Body mass index tracked strongly during adolescence, suggesting that interventions need to start as early as possible. Dumith et al provide more information on the predictors and health consequences of screen time during adolescence. Screen time increased from 11 to 15 years of age, particularly among boys and high-income adolescents [9]. This increase in screen time predicted adiposity in this cohort. And in a qualitative contribution to this supplement, Gonçalves et al report new insights on obesity from a subsample of the birth cohort. Low-income obese adolescents and their mothers perceive obesity as a “heritage” caused by family genes, the side effects of medication use, and stressful life events, whereas low-income eutrophic families emphasize the role of an unhealthy diet in the development of obesity [10]. Among high-income families, those who are obese “blame” genetic factors and emotional problems, whereas those who are nonobese again cite the role of diet and physical inactivity as key factors [10].

The supplement closes with a study showing an alarmingly prevalence of self-medication (29%) among 15-year-old adolescents, with girls being more likely to self-medicate than boys [11]. Use of medicines in early childhood predicts future use, and previous self-medication predicts future self-medication. Documenting this early trajectory of medicine use provides an opportunity to plan preventive interventions that promote the rational use of medicines by families and their children.

This collection of articles examining the Pelotas cohort provides us with a rare opportunity to understand clearly the critical role of a life-course perspective in promoting the health and well-being of adolescents. We look forward to seeing these findings replicated in other settings, but the results presented here are robust and have relevance to populations throughout the world. Consider the collective critical findings:

∘ First, several factors arise early in development. Further, the behaviors and social context of the family affects not just the young child but also the adolescent [1–4,11].
∘ Second, behaviors tend to track from childhood to adolescence, suggesting once again that interventions need to start earlier, preferably before individuals get on the wrong track [3,4,9–11].
∘ Third, some exposures (e.g., physical activity) might have both benefits (lung function) and harmful effects (school failure), depending on the dose [5,6].
∘ Fourth, qualitative studies provide us with an opportunity to hear the nuances that come from adolescents and families that are difficult to extract from quantitative studies [10].

Although these studies collectively provide valuable insights into child development, it is important to note that the Pelotas cohort only provides us with information through mid-adolescence (15 years). Whereas it is clear that many of the behaviors and health outcomes examined in these studies have their roots in early childhood, the brain continues to develop well into young adulthood [12]. Although the articles in this supplement point to the critical importance of early preventive interventions, late adolescence and young adulthood also provide opportunities to continue promoting the healthy development of the adolescent.
